# Gastric Tube Volvulus Occurring Years After Esophagectomy and Its Successful Treatment Via Endoscopic Stenting

**DOI:** 10.14309/crj.0000000000001435

**Published:** 2024-07-17

**Authors:** Alexander Miller, Rohitha Moudgal, Obaida Dairi, Jeffrey M. Adler, Richard I. Rothstein

**Affiliations:** 1 Department of Medicine, Dartmouth Health, Lebanon, NH; 2 Department of Vascular Medicine, Cleveland Clinic, Cleveland, OH; 3 Geisel School of Medicine at Dartmouth, Hanover, NH; 4 Dartmouth Health Department of Gastroenterology and Hepatology, Lebanon, NH

**Keywords:** esophagectomy, gastric tube volvulus, endoscopic stenting

## Abstract

Esophageal cancer is frequently treated with esophagectomy, which is associated with distinct complications. Delayed gastric conduit emptying is a well-recognized complication that usually occurs within the postoperative period. By contrast, gastric tube volvulus is a rarer complication with a more variable time course of onset after esophagectomy and can be mistaken for delayed gastric conduit emptying. We describe the fifth reported case of gastric tube volvulus occurring years after esophagectomy and its successful treatment via endoscopic stenting.

## INTRODUCTION

Esophageal cancer is a major cause of cancer-related death worldwide.^1^ Esophagectomy is a mainstay of treatment for patients with resectable locoregional disease.^2^ Delayed gastric conduit emptying (DGCE) is a common complication noted after surgery, occurring in approximately 10%–20% of patients.^3–6^ These patients present days after surgery with symptoms of gastric outlet obstruction, and the pathophysiology is believed to be related to surgical denervation.^3^ For treatment, patients with DGCE routinely undergo therapeutic endoscopic pyloric dilation.^4–6^ By contrast, gastric tube volvulus is a relatively rare complication of esophagectomy and previously has been reported in only 4 case reports.^7–10^ Patients with gastric tube volvulus typically present symptoms similar to DGCE and the time of onset ranges from weeks to years after surgery.^7–10^ Previous treatments have included stent placement, as well as endoscopic reduction and gastropexy.^7–10^ We describe the fifth reported case of gastric tube volvulus, including its diagnosis by barium swallow and treatment via endoscopic stent placement.

## CASE REPORT

Three years after undergoing Ivor-Lewis esophagectomy for T1b adenocarcinoma of the esophagus, a 66-year-old transgender woman presented to her thoracic surgeon with weight loss, significant dyspepsia, and regurgitation after meals. Chest computed tomography imaging showed an “ill-defined soft tissue thickening in the upper retroperitoneum interposed between gastric pull-through and the celiac axis.” She subsequently underwent flexible esophagoscopy, which found food debris within the gastric conduit but did not reveal evidence of recurrence of her adenocarcinoma. Although the endoscope easily traversed the pylorus, her symptoms were presumed to be due to DGCE, and her thoracic surgeon performed balloon pyloric dilation.

In the 3 months following this attempted endoscopic intervention, the patient experienced worsening of her gastric outlet obstruction symptoms associated with severe weight loss. After multiple emergency department visits, inpatient admission to thoracic surgery's hospital service, and thoracic surgery clinic appointments, her providers seemingly continued to attribute her symptoms to DGCE. She underwent esophagogastroduodenoscopy, with repeat balloon pyloric dilation and placement of a jejunostomy feeding tube. This procedure did not alleviate her symptoms.

**Figure 1.**
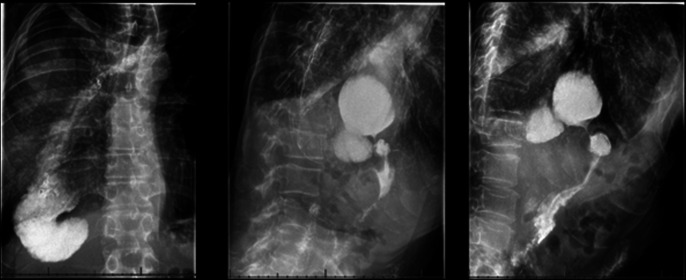
Images from X-ray barium swallow with contrast showing widely patent esophagogastric anastomosis, redundant intrathoracic portion of the proximal gastrointestinal tract, and a narrowed lumen at the level of the diaphragm.

Four months after the initial onset of her symptoms, the patient presented as a new patient to a gastroenterology clinic for continued worsening of her symptoms causing failure to thrive. A barium swallow study found a redundant intrathoracic portion of the proximal gastrointestinal tract with a tight focal narrowing causing obstruction (Figure 1). She subsequently underwent upper endoscopy to evaluate for suspected gastric outlet obstruction. This procedure visualized 2 L of gastric fluid proximal to a benign-appearing pinch of gastric folds, approximately 5 cm proximal to a normal-appearing pylorus. This gastric tube volvulus was traversed with gentle pressure and treated with an 18 × 59 mm covered metal stent, which was secured using two 2.0 polypropylene sutures with clinches on both ends using the Apollo endosuture technique. No evidence of extraluminal compression was seen with esophagogastroduodenoscopy or by endoscopic ultrasound.

**Figure 2.**
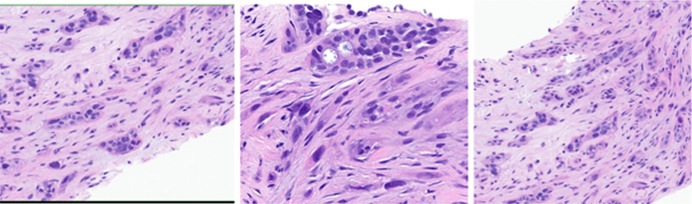
Pathology slides from sternal core biopsy showing the architecture (left hand frame), glandular component (middle frame), and the squamous component (right hand frame) of the adenosquamous carcinoma of unclear origin.

Following this procedure, the patient found immediate relief from her symptoms and no longer required the use of her jejunostomy feeding tube. A positron emission tomography computed tomography scan was obtained to evaluate for the possibility of adenocarcinoma recurrence causing external compression of the gastric conduit. Although this scan was without evidence of intrathoracic/intra-abdominal lesions, there were unexpectedly innumerable focal skeletal lesions, suggesting new metastatic disease. The patient underwent sternal bone biopsy, and pathology was most consistent with adenosquamous carcinoma of unclear origin on immunohistochemistry (Figure 2). Repeat endoscopy 1 month later demonstrated patency of her gastric conduit stent, and she was tolerating oral alimentation. The patient was subsequently referred to both oncology and palliative care clinics and died approximately 7 months after the initial onset of her symptoms.

## DISCUSSION

Gastric tube volvulus is a rare but distinct cause of gastric content retention. We hypothesize that this patient's gastric tube volvulus initially began when weight loss—secondary to recurrent metastatic esophageal adenocarcinoma—caused the appearance of longitudinally redundant gastric conduit, which then formed an obstructive folding. To the best of our knowledge, this is the fifth documented case of a gastric tube volvulus occurring after esophagectomy. Our case report is most similar to the case recently reported by Schizas et al, in that both cases occurred following non-total esophagectomy.^7^ By contrast, the other 3 cases of gastric tube volvulus in the available literature all occurred following total esophagectomy.^8–10^ The patient described within our case report was treated via placement of covered metal stent within the volvulus, which is similar to the treatment strategies used by prior authors.^9,10^ Other preceding reports include successful gastropexy to treat gastric tube volvulus, as well as endoscopic reduction.^7,8^ Given that the patient featured in our case report had immediate and sustained resolution of symptoms, it appears that endoscopic stent placement is an effective treatment option for gastric tube volvulus.

In conclusion, gastric tube volvulus is a rare complication of esophagectomy that can mimic the more common symptoms of DGCE. Owing to its similarity to DGCE, gastric tube volvulus may be misdiagnosed, resulting in a delay in its treatment. Diagnosis via contrast imaging and endoscopy is vital to the recognition and treatment of postsurgical gastric tube volvulus.

## DISCLOSURES

Author contributions: A. Miller made substantial contributions to drafting, editing, and collecting the information; R. Moudgal: conception and acquisition of data; O. Dairi: design of work, reviewing for important content; JM Adler: acquisition of data, reviewing the work, final approval and agreement; RI Rothstein: conception, design, acquisition, interpretation of work, drafting and reviewing, patient consent and agreement for appropriate investigation. All authors gave final approval and agreed to be held accountable for the work. RI Rothstein is the article guarantor.

Financial disclosure: None to report.

Informed consent was obtained for this case report.
